# Acute effect of *Clitoria ternatea* flower beverage on glycemic response and antioxidant capacity in healthy subjects: a randomized crossover trial

**DOI:** 10.1186/s12906-017-2075-7

**Published:** 2018-01-08

**Authors:** Charoonsri Chusak, Thavaree Thilavech, Christiani Jeyakumar Henry, Sirichai Adisakwattana

**Affiliations:** 1 Department of Nutrition and Dietetics, Faculty of Allied Health Sciences, Chulalongkorn University, 154 Rama 1 Rd., Wangmai, Pathumwan, Bangkok, 10330 Thailand; 2 Clinical Nutrition Research Centre (CNRC), Singapore Institute for Clinical Sciences (SICS), Agency for Science, Technology and Research (A*STAR), Singapore, Singapore; 3 Department of Biochemistry, Yong Loo Lin School of Medicine, National University of Singapore, Singapore, Singapore

**Keywords:** *Clitoria ternatea*, Glucose, Insulin, Antioxidant, Glycemia, Sucrose

## Abstract

**Background:**

*Clitoria ternatea* L., a natural food-colorant containing anthocyanin, demonstrated antioxidant and antihyperglycemic activity. The aim of this study was to determine the effects of *Clitoria ternatea* flower extract (CTE) on postprandial plasma glycemia response and antioxidant status in healthy men.

**Methods:**

In a randomized, crossover study, 15 healthy men (ages 22.53 ± 0.30 years; with body mass index of 21.57 ± 0.54 kg/m^2^) consumed five beverages: (1) 50 g sucrose in 400 mL water; (2) 1 g CTE in 400 mL of water; (3) 2 g CTE in 400 mL of water; (4) 50 g sucrose and 1 g CTE in 400 mL of water; and (5) 50 g sucrose and 2 g CTE in 400 mL of water. Incremental postprandial plasma glucose, insulin, uric acid, antioxidant capacities and lipid peroxidation were measured during 3 h of administration.

**Results:**

After 30 min ingestion, the postprandial plasma glucose and insulin levels were suppressed when consuming sucrose plus 1 g and 2 g CTE. In addition, consumption of CTE alone did not alter plasma glucose and insulin concentration in the fasting state. The significant increase in plasma antioxidant capacity (ferric reducing ability of plasma (FRAP), oxygen radical absorbance capacity (ORAC), trolox equivalent antioxidant capacity (TEAC), and protein thiol) and the decrease in malondialdehyde (MDA) level were observed in the subjects who received 1 g and 2 g CTE. Furthermore, consumption of CTE protected sucrose-induced reduction in ORAC and TEAC and increase in plasma MDA.

**Conclusions:**

These findings suggest that an acute ingestion of CTE increases plasma antioxidant capacity without hypoglycemia in the fasting state. It also improves postprandial glucose, insulin and antioxidant status when consumed with sucrose.

**Trial registration:**

Thai Clinical Trials Registry: TCTR20170609003. Registered 09 September 2017.

‘retrospectively registered’.

## Background

Non-communicable diseases (NCDs) are a leading cause of death across all age groups and remain a global health problem worldwide. The NCDs deaths are projected to rise by 20% in Southeast Asia, a sub-region of Asia [1]. The development of NCDs are commonly associated with modifiable behavioral risk factors; for example: unhealthy diets, physical inactivity, alcohol and tobacco use and non-modifiable risk factors such as age, gender or family history [2]. These risk factors play an important role in metabolic/physiological changes, which contribute to the development of overweight/obesity and hyperglycemia [2, 3]. A recent study showed that consumption of sugar-rich diets has been associated with increased postprandial glucose level [4]. Furthermore, the rise in postprandial glucose directly induces the production of mitochondrial reactive oxygen species (ROS) through glycolysis pathway, polyol pathway and glycation [5, 6]. Subsequently, ROS depletes antioxidant enzyme activities that are responsible for causing several pathologies of health implications [7].

Several studies have shown that the reduced rate of carbohydrate absorption and digestion is a significant strategy to suppress postprandial hyperglycemia by inhibition of carbohydrate digestive enzymes such as pancreatic α-amylase and intestinal α-glucosidase [8–10]. Interestingly, plant bioactive compounds such as polyphenols and anthocyanins have been shown to inhibit pancreatic α-amylase and α-glucosidase activity [10, 11]. For example, phenolic-enriched black rice flour extract delayed starch hydrolysis by inhibiting carbohydrate digestive enzymes [12]. After consumption of berry puree with sucrose, the amplitude and the peak postprandial plasma glucose were significantly lower than the consumption of sucrose alone in healthy subjects [13]. Furthermore, consumption of plant-based antioxidants improved plasma antioxidant capacity in human subjects [14]. It has been demonstrated that consumption of berries, apples, mixed grapes or kiwifruits was associated with increased postprandial plasma antioxidants including oxygen radical absorbance capacity (ORAC) and ferric-reducing ability of plasma (FRAP) [15–17]. Therefore, plant-based antioxidant may be considered as a rich source of natural antioxidants for protecting postprandial oxidative stress.

*Clitoria ternatea* L., commonly known as Butterfly pea, is a plant species belonging to the Fabaceae family. This plant is widely distributed in tropical zones such as Asia, the Caribbean, Central and South America. *Clitoria ternatea* has been extensively used as a traditional herbal medicine. In addition to biological activities, *Clitoria ternatea* flower is a source of natural blue food and beverage colorant worldwide [18]. Previous studies have reported the pharmacological properties of *Clitoria ternatea* including antiplatelet aggregation, vasodilation, antidiabetic, and antioxidant activity [19]. It was recently reported that aqueous *Clitoria ternatea* flower extract inhibited carbohydrate digestive enzymes such as intestinal α-glucosidase and pancreatic α-amylase in vitro [11]. To enhance bioaccessibility, microencapsulation of *Clitoria ternatea* flower extract increased the ability to inhibit pancreatic α-amylase and antioxidant activity after simulated gastrointestinal digestion [20]. Although the bioactivities of *Clitoria ternatea* flower extract (CTE) are well-documented, clinical studies addressing the impact of CTE on glycemic response and antioxidant capacity remain unknown. Therefore, the aim of this study was to determine the effects of CTE on postprandial glycemic response and antioxidant status in healthy subjects.

## Methods

### Plant preparation and extraction

The *Clitoria ternatea* flower was purchased from a local herbal drug store, Bangkok, Thailand. The herbarium number of *Clitoria ternatea* was authenticated at the Princess Sirindhorn Plant Herbarium, Plant Varieties Protection Division, Department of Agriculture, Bangkok, Thailand, Voucher specimen: BKU066793. The flowers were dried and then boiled twice with distilled water in 1:20 *w*/*v* ratio at 90–95°C for 4 h. After filtering with Whatman No.1, the aqueous extract was dried by using a spray dryer SD-100 (Eyela world, Tokyo Rikakikai Co., LTD, Japan) with a specific condition including inlet temperature at 178 °C, outlet temperature at 85–95°C, blower at 0.60–0.65 m^3^/min and atomizing at 90 kPa. The powder of *Clitoria ternatea* flower extract (CTE) was immediately kept in a laminated aluminum foil vacuum bag at room temperature before use. The CTE was evaluated for phenolic content by the Folin-Ciocalteau method [21] and total anthocyanin contents by the pH differential method with minor modifications [22]. Total phenolic compounds and anthocyanins in CTE were 53.08 ± 0.08 mg gallic acid equivalents/ g extract and 1.08 ± 0.12 mg delphinidin-3-glucoside equivalents /g extract.

### Participants

The sample size was calculated according to Vuksan et al. [23], considering the incremental area under the curve (iAUC) of postprandial glucose response as the main variable. A statistical power of 80% and an expected difference of 21% in the baseline values were adopted to form a total sample of at least 13 individuals. Eighteen healthy men were screened and recruited by advertisement at Chulalongkorn University. Inclusion criteria were 20–40 years old men, body mass index (BMI) in the range of 18.5–22.9 kg/m^2^, fasting plasma glucose <100 mg/dL, fasting plasma triglyceride <150 mg/dL, total cholesterol concentration < 200 mg/dL, blood urea nitrogen (BUN) in range of 5–20 mg/dL, creatinine in range of 0.6–1.2 mg/dL, AST < 40 U/L, ALT <40 U/L and non-smokers and non-alcohol users. Participants were excluded from the study if they were diagnosed with any chronic diseases such as diabetes mellitus etc. or took any medication or used any dietary supplements related to antioxidants and glycemic response. They were free to withdraw from the study at any time.

### Ethics

The study protocol was approved by the office of Ethics Review Committee for Research Involving Human Research Subjects, Human Science Group, Chulalongkorn University (COA No. 187/2558 and No.061/2560). All subjects gave their written informed consent to participate. All information of participants was kept confidential. There were no major changes in the study protocol after initiation of the study. The baseline characteristics of participant are described in Table 1.

**Table 1:** Participant characteristics

	Mean ± SEM
Age (years)	22.53 ± 0.30
Height (cm)	173.65 ± 1.46
Weight (kg)	65.16 ± 2.05
BMI (kg/m^2^)	21.57 ± 0.54
Fasting plasma glucose (mg/dL)	84.77 ± 1.94
Total cholesterol (mg/dL)	183.22 ± 6.29
Serum triglyceride (mg/dL)	81.00 ± 6.23
LDL-C (mg/dL)	120.56 ± 6.44
Creatinine (mg/dL)	0.95 ± 0.04
BUN (mg/dL)	10.53 ± 0.48
ALT (U/L)	21.55 ± 0.99
AST (U/L)	15.27 ± 2.35
Systolic blood pressure (mmHg)	115.54 ± 1.42
Diastolic blood pressure (mmHg)	68.21 ± 1.51

All values are means ± SEM, n = 15

### Study design and protocol

The study was randomized with 5 crossover trials with 1 week of washout period. The washout period in this study considered as a duration of intervention and our previous experiments [24]. The participants were randomly assigned to 1 of 5 intervention drinks. Randomization was achieved by a researcher using a computer to generate random numbers, simple randomization was used. Randomization numbers were assigned to participants after their screening assessments. Treatment allocation occurred when the participants met the inclusion criteria and signed the informed consent form. Randomization sequence and allocation was concealed to all study participants until completion of the study. On the test day, the participants were asked to fast for 10–12 h overnight and avoid consumption of phytochemical-rich foods such as tea, berries, soy, red grape and orange etc. starting 3 days before each intervention period until completion of the study. After participants arrived at the study, the participants were seated for 10 min to rest. An intravenous catheter was then inserted into a peripheral arm vein for repeated blood collection by a registered nurse. After collection of blood for baseline, an assigned drink was consumed within 5 min. The subjects consumed five different beverages: (1) 50 g sucrose in 400 mL water; (2) 1 g CTE in 400 mL of water; (3) 2 g CTE in 400 mL of water; (4) 50 g sucrose and 1 g CTE in 400 mL of water; and (5) 50 g sucrose and 2 g CTE in 400 mL of water. All beverages were freshly prepared and packaged in identical containers at the kitchen at Chula 3 building, Faculty of Allied Health Sciences before distribution to participants, and numbered sequentially according to randomization schedule. The beverage quality controls during the preparation process was done by quantitative measurement of total polyphenols and anthocyanins following the methods mentioned previously. The beverages have been coded, participants were blind to the randomization sequence and treatment allocation until the completion of the study. No other food or drink was allowed after consuming the beverage. Blood collection was performed at intervals over 180 min before and after administration of the beverage. Blood samples were centrifuged at 3000 rpm for 10 min at 4 °C and the plasma samples were then kept at −20 °C before analysis. The plasma samples were analysed for glucose, insulin, ferric reducing ability of plasma (FRAP), oxygen radical absorbance capacity (ORAC), trolox equivalent antioxidant capacity (TEAC), thiol, and malondialdehyde (MDA).

### Determination of plasma glucose, insulin and uric acid

Plasma glucose was determined by using a glucose oxidase method (HUMAN GmbH, Germany). The Human Insulin ELISA kit was used for the quantitative determination of plasma insulin (GenWay Biotech Inc., San Diego, CA, USA). The Enzymatic Colorimetric Teat for Uric Acid with Lipid Clearing Factor (LCF) was performed to measure plasma uric concentration (HUMAN GmbH, Wiesbaden, Germany).

### Determination of plasma antioxidant capacity

The plasma FRAP level was analyzed according to a previous report [25]. The plasma sample (10 μL) was mixed with FRAP reagent (90 μL) containing 0.3 M sodium acetate buffer (pH 3.6), 10 mM TPTZ in 40 mM HCl and 20 mM FeCl_3_. After incubation in a dark room maintained at room temperature for 30 min, the reaction was measured at the absorbance at 595 nm. Plasma FRAP level was reported as FeSO_4_ equivalents. The plasma ORAC level was determined according to a previous study [26]. The plasma sample (25 μL) was incubated with 4.8 nM sodium fluorescein in 75 mM PBS at 37 **°**C for 10 min. Then, 64 mM AAPH solution was added and the mixture was measured the fluorescence intensity (λ_excitation_ = 485 mM and λ_emission_ = 535 nm) every 2 min for 1 h. The ORAC value was calculated from the calibration curve of net AUC against Trolox concentration and expressed as μM Trolox equivalent. The plasma trolox equivalent antioxidant capacity (TEAC) level was determined according to a previous study (24). The 2,2′-azinobis(3-ethylbenzothiazoline-6-sulfonate) free radical (ABTS^●+^) solution was prepared by the mixture of 7 mM ABTS in 0.1 M PBS (pH 7.4) and 2.45 mM K_2_S_2_O_8_ in distilled water (1,1, *v*/v) [27]. After 16 h of the incubation at room temperature in the dark, ABTS^●+^ solution was diluted with 0.1 M PBS (pH 7.4) to adjust the absorbance between 0.900 and 1.000 at 734 nm. The adjusted ABTS^●+^ solution was added in the 1:5 diluted plasma and then incubated for 6 min. The reaction was measured at 734 nm and plasma TEAC was expressed as mM Trolox equivalents. The plasma thiol group level was measured using an Ellman’s assay [28] with slightly modification. The plasma sample (90 μL) was mixed with 2.5 mM 5,5′-dithiobis-(2-nitrobenzoic acid) (DTNB) in 0.1 M PBS (pH 7.4). After incubation at room temperature for 15 min, the reaction was measured the absorbance at 410 nm. The plasma thiol level was calculated and expressed as μM L-cysteine equivalent. The plasma MDA level was determined according to a previously described report [29]. Plasma malondialdehyde (MDA) was quantified using a method based on the formation of thiobarbituric acid reactive substances (TBARS). The plasma sample (150 μL) was mixed with 10% thichloroacetic acid (TCA) and 50 mM 2,6-di-tert-butyl-4-methylphenol (BHT). After centrifugation at 13000 rpm at 4 **°**C for 10 min, 0.67% thiobarbituric acid (TBA) was added to the supernatant. The mixture was heated at 95 **°**C for 10 min and measured the absorbance at 532 nm. The plasma MDA level was expressed as nmol/L MDA equivalent.

### Statistical analyses

The results were reported as mean ± SEM. For each test, the incremental data of plasma glucose, insulin, and antioxidant were analyzed by using a Kolmogorov-Smirnov test for normality testing. Statistical analysis of the incremental data was carried out using two-way repeated-measures (two-factor repetition) ANOVA, with beverage and time as within-subject factors. The Duncan post hoc comparison (*P* < 0.05) was utilized to assess both mean differences among the beverages within a single time point and intervention effects at different time points within the treatments with respect to baseline. The incremental area under the curve (iAUCs) was calculated by using according to the trapezoidal method. Then, one-way repeated measures ANOVA with Duncan post hoc comparison (*P* < 0.05) was evaluated.

## Results

### Subjects

**Fig. 1:**
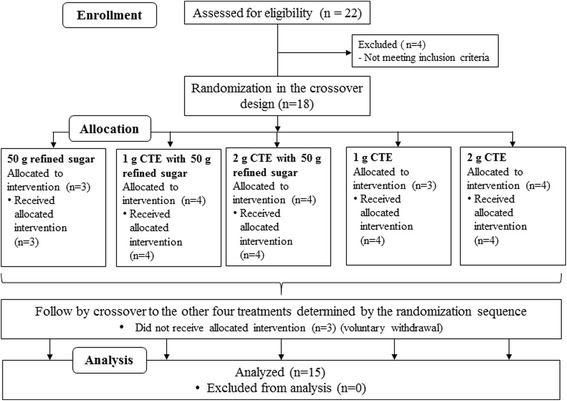
The flowchart describing the trial

Between October 2015 and January 2017, twenty-two subjects were recruited for this study according to the flow chart (Fig.1). Four subjects were excluded from the study as they did not meet the inclusion criteria of the study. The eighteen remaining subjects were randomly assigned into 5 groups. Three subjects withdrew during the study due to the reasons unrelated to the study. Fifteen subjects finally completed the study. The baseline characteristics of the fifteen subjects are shown in Table 1. No adverse events after consumption of beverages were observed.

### Plasma glucose, insulin and uric acid concentration

**Fig. 2:**
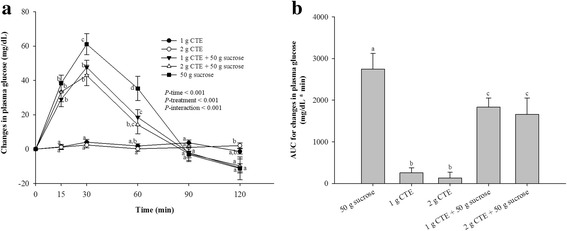
Postprandial plasma glucose response of five different beverages. **a** Changes in glucose concentrations during 120 min of five different beverages; (**b**) Glucose changes area under the curves (AUCs) of five different beverages. Values are means ± SEM, *n* = 15. Different letters are significantly different (*P* < 0.05)

The results of postprandial glucose concentration are presented in Fig. 2. At the individual time points, consumption of 1 and 2 g CTE did not significantly change in the baselines of glucose concentration. Consumption of sucrose caused a rapid rise of glucose, with the peak concentration at 30 min, followed by a rapid fall below the baseline level within 120 min. Ingestion of 1 g and 2 g CTE together with sucrose significantly decreased postprandial plasma glucose concentration at 30 and 60 min (*P* < 0.05). As shown in Fig. 2b, 1g and 2 g CTE plus sucrose resulted in 0.67-and 0.60-fold lower iAUCs for plasma compared with sucrose, respectively.

**Fig. 3:**
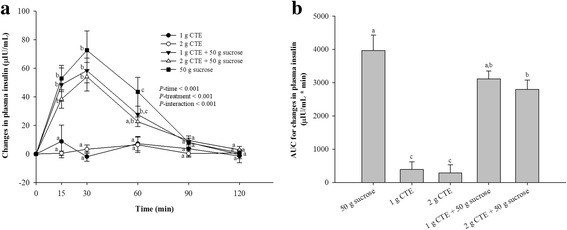
Postprandial plasma insulin response of five different beverages. **a** Changes in insulin concentrations during 120 min of five different beverages; (**b**) Insulin changes area under the curves (AUCs) of five different beverages. Values are means ± SEM, n = 15. Different letters are significantly different (*P* < 0.05)

As shown in Fig. 3a, there was significant effect on plasma insulin concentration for time, treatment and time x treatment interaction (*P* < 0.001). The results demonstrated that consumption of 1 g and 2 g CTE did not alter the plasma insulin concentration. After consumption of sucrose, the peak of plasma insulin concentration was observed at 30 min and returned to the baseline within 90 min. The results showed that only consumption of 2 g CTE with sucrose significantly suppressed a rise in postprandial plasma insulin at 60 min compared to sucrose (*P* < 0.05). In Fig. 3b, the iAUCs for plasma insulin of 2 g CTE plus sucrose were 0.67 times less than that of sucrose. However, the iAUCs for plasma insulin had no significant difference between 1 g and 2 g of CTE without sucrose.

**Fig. 4:**
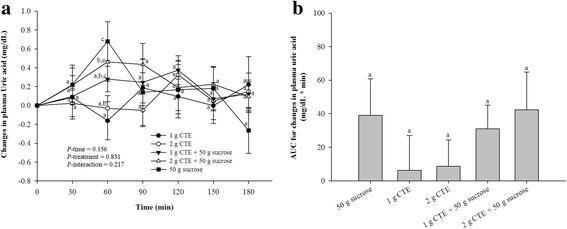
Postprandial plasma uric acid of five different beverages. **a** Changes in uric acid concentrations during 180 min of five different beverages; (**b**) Uric acid changes area under the curves (AUCs) of five different beverages. Values are means ± SEM, n = 15. Different letters are significantly different (*P* < 0.05)

Our results demonstrated that postprandial plasma uric acid increased rapidly at 60 min after ingestion of sucrose. No main effects for time, treatment and their interactions were observed. In contrast, CTE ingestion with or without sucrose did not significantly increase plasma uric acid (Fig. 4a). Postprandial iAUCs for plasma uric acid did not differ between all beverages, as shown in Fig. 4b.

### Plasma antioxidant status

**Fig. 5:**
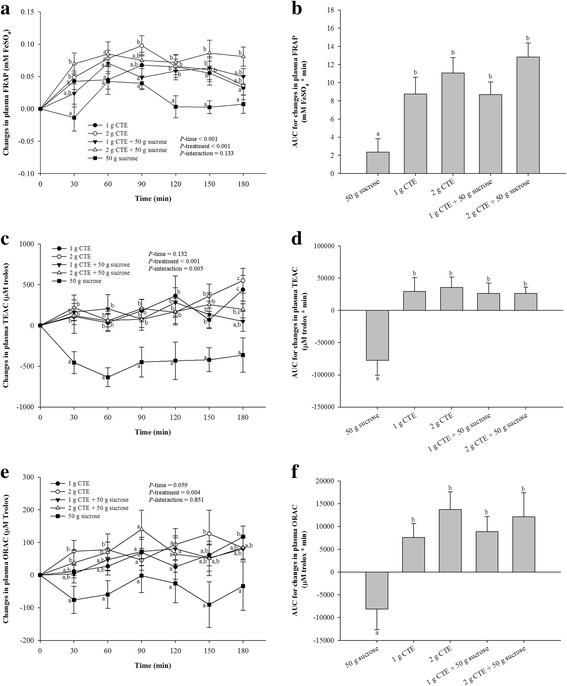
Postprandial plasma antioxidant capacities of five different beverages. **a** Changes in FRAP, (**c**) TEAC and (**e**) ORAC concentrations during 180 min of five different beverages; (**b**) FRAP, (**d**) TEAC and (**f**) ORAC changes area under the curves (AUCs) of five different beverages. Values are means ± SEM, n = 15. Different letters are significantly different (*P* < 0.05)

Figure 5a shows the changes in postprandial plasma FRAP level after consumption of CTE. The postprandial plasma FRAP was slightly increased after 60 min consumption of sucrose. In addition, 1 g and 2 g CTE plus sucrose showed a maximum peak at 60 min, whereas the significant results were observed at 120 and 150 min when compared to sucrose group (*P* < 0.05). In contrast, 1 g and 2 g CTE could increase the postprandial plasma FRAP at 30 min and maintained its effect throughout the postprandial period. When compared to the group which received sucrose, the iAUCs for plasma FRAP were significantly higher 3.71- and 5.48-fold after consumption of sucrose with 1 g and 2 g CTE, respectively (Fig. 5b). Furthermore, consumption of 1 g and 2 g CTE were 3.74 and 4.73-fold higher iAUCs for plasma FRAP than that of sucrose.

As shown in Fig. 5c, ingestion of sucrose showed significantly lower postprandial plasma TEAC than 1 g and 2 g CTE with or without sucrose during the postprandial period (*P* < 0.05). Compared with sucrose, iAUCs for plasma TEAC after consumption 1 g and 2 g CTE with sucrose were both significantly higher 1.34-fold (Fig. 5d). Additionally, iAUCs for plasma TEAC was 1.38- and 1.46-fold higher after 1 g and 2 g CTE, respectively.

The decreased postprandial plasma ORAC was seen after 30 min of sucrose ingestion (Fig. 5e). By contrast, the reduction in plasma ORAC was suppressed after CTE consumption compared with that of sucrose. Furthermore, Fig. 5f shows the iAUCs for plasma ORAC after consumption CTE. After ingestion of 1 g and 2 g CTE with sucrose, iAUCs for plasma ORAC were 2.09- and 2.49-fold significantly increased compared with that in the sucrose. The iAUCs for plasma ORAC after 1 g and 2 g CTE were 1.93- and 2.69-fold, respectively (*P* < 0.05).

**Fig. 6:**
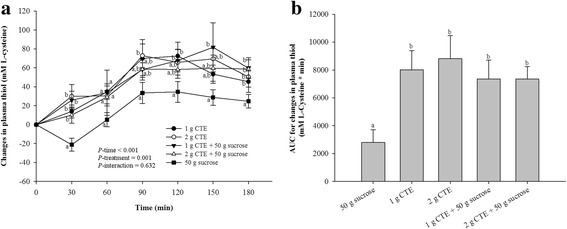
Postprandial plasma thiol of five different beverages. **a** Changes in plasma thiol concentrations during 180 min of five different beverages; (**b**) Thiol changes area under the curves (AUCs) of five different beverages. Values are means ± SEM, n = 15. Different letters are significantly different (*P* < 0.05)

Postprandial plasma thiol level was significantly reduced at 30 min after sucrose consumption as shown in Fig. 6a. A significant effect for treatment (*P* = 0.001) and time (*P* < 0.001) was observed. Consumption of 1 g and 2 g CTE with or without sucrose caused a significant rise in postprandial plasma total thiol at 30 and 180 min after (*P* < 0.05). The iAUCs for plasma thiol concentration after CTE consumption were significantly different from that with sucrose (Fig. 6b). There were 2.87- and 3.15-fold significantly higher after 1 g and 2 g CTE, respectively. The iAUCs for plasma thiol after both 1 g and 2 g CTE with sucrose were resulted in 2.63-fold higher than the corresponding iAUCs after sucrose (*P* < 0.05).

### Lipid peroxidation

**Fig. 7:**
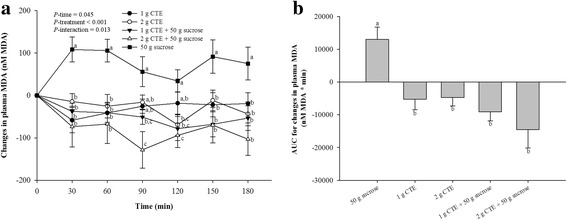
Postprandial plasma MDA of five different beverages. **a** Changes in MDA concentrations during 180 min of five different beverages; (**b**) MDA changes area under the curves (AUCs) of five different beverages. Values are means ± SEM, n = 15. Different letters are significantly different (*P* < 0.05)

The postprandial plasma MDA significantly increased after 30, 60, 150 and 180 after ingestion of sucrose (Fig. 7a). The results showed that 1 g and 2 g CTE with sucrose significantly reduced plasma MDA at all individual time points (*P* < 0.05). Ingestion 1 g and 2 g CTE also reduced plasma MDA at 30, 60, 150 and 180 min (*P* < 0.05). There were significant lower in iAUCs for plasma MDA after CTE consumption with and without sucrose (Fig. 7b).

## Discussion

Consumption of foods and beverages containing high sucrose markedly induces postprandial hyperglycemia. This effect may result in overstimulation of insulin from pancreatic β-cells causing hyperinsulinemia [30]. Recently, hyperinsulinemia has been associated with the development of metabolic syndromes and gestational and type 2 diabetes [31]. Furthermore, postprandial hyperglycemia and hyperinsulinemia play an important role in excessive generation of reactive oxygen species (ROS) such as hydrogen peroxide, hydroxyl radicals and superoxide anion [31, 32]. Overproduction of ROS predominates the endogenous antioxidant capacity, causing imbalance of antioxidative defenses and consequently oxidative damage to the protein, lipids and DNA [33]. Postprandial hyperglycemia can be controlled by retarding the absorption of glucose through inhibition of intestinal sucrase, the rate-limiting enzyme in the conversion of sucrose to glucose and fructose before absorption [34]. Previously, consumption of blackcurrants and lingonberries suppressed postprandial rise of plasma glucose in healthy participants compared to sucrose reference due to the inhibitory effect of berries on α-glucosidase activity [35]. The delayed digestion of sucrose and absorption of glucose after berries consumption contributed to lower postprandial insulin response. In addition, the reduced hyperinsulinemia has been associated with reduced the risk of insulin resistance, endothelial dysfunction, obesity, and metabolic syndromes [32, 36]. Our findings showed the suppression of the peak postprandial glucose and insulin concentration after consumption of CTE and sucrose. Previous findings indicate that CTE inhibited carbohydrate digestive enzymes such as intestinal α-glucosidase and pancreatic α-amylase in vitro [11]. It suggests that CTE suppresses sucrose-induced postprandial glucose and insulin responses through the inhibition of intestinal sucrase. It is noted that phenolic compounds in edible plants can inhibit pancreatic α-amylase and α-glucosidase, resulting in the delay of postprandial glucose [37]. Major phenolic compounds in CTE composed of anthocyanins including delphinidin-3,5-glucoside, delphinidin-3-glucoside, malvidin-3β-glucoside, kaempferol, *p*-coumaric acid and six major ternatins (ternatins A1, A2, B1, B2, D1 and D2). These compounds could inhibit pancreatic α-amylase and α-glucosidase activity [38]. For example, delphinidin-3-glucoside and kaempferol showed the inhibitory effect against pancreatic α-amylase and α-glucosidase activity in vitro [39, 40] which may be useful as a potential inhibitor for delaying postprandial hyperglycemia. We suggest that the phenolic compounds in CTE may contribute to delay the hydrolysis of sucrose to glucose and fructose by inhibiting intestinal sucrase. However, the biological action of CTE may be result of other classes of phytochemical compounds. In the further study, isolation of individual phytochemical constituents is needed to investigate intestinal α-glucosidase and pancreatic α-amylase inhibitory activity. Furthermore, we found that CTE alone did not alter postprandial glucose and insulin level, indicating that antihyperglycemic activity of CTE is not involved in the insulin secreting activity. Thus, drinking CTE may not produce hypoglycemia in the fasting state.

Reactive oxygen species (ROS) can be normally generated from nutrient metabolisms consumption of carbohydrate-containing foods and beverages [41]. Increased production of ROS contributes to an imbalance condition between oxidative generation and antioxidant defense, resulting in a consequence of postprandial oxidative stress [42]. Thiols, also called sulfhydryls, exist in proteins in the side-chain of cysteine (Cys) amino acids [43]. Albumin, the most abundant proteins, plays a major role in the total defense system against free radicals and oxidative damage in plasma through enzymatic and non-enzymatic mechanisms [43, 44]. A previous study reported that a high sucrose intake resulted in reduced antioxidant defense mechanisms and increased oxidative stress [44]. The reduced plasma thiols were markedly observed in the participants receiving high carbohydrate diet (69% of total energy) [24]. Malondialdehyde (MDA), a marker of lipid peroxidation, is produced from the oxidation process of polyunsaturated fatty acids (PUFA) in the cell membrane. During unstable free radicals promoting chain reaction, double bounds of PUFA are cleaved and then released as bis-aldehyde MDA. Overproduction of ROS has shown the positive correlation with the production of MDA [45]. Moreover, excessive intake of sucrose provokes the generation of peroxidation markers concomitant with reduced plasma antioxidant capacities.

Our findings are consistent with previous studies that reduced total antioxidant capacity concomitant with increased plasma MDA was detected after consumption of sucrose beverage [46, 47]. The various methods have been utilized for the measurement of total antioxidant capacity; TEAC assay has been used for the assessment of antioxidant status to scavenge the ABTS radical cation compared to Trolox, a water soluble analogue of vitamin E; FRAP assay based on the reduction of ferric-TPTZ complex to its ferrous for the measurement of total reducing power of antioxidants; and ORAC assay measures the water-soluble antioxidants inhibition of peroxyl radical which can induce oxidations and thus reflects classical radical chain breaking antioxidant activity by hydrogen atom transfer [16]. From our results, the observed postprandial alterations of antioxidant capacity were markedly attenuated when receiving sucrose and CTE. Interestingly, CTE could increase plasma antioxidant capacity and maintain the level of thiol group and subsequently reduced plasma MDA concentration. Several studies have shown the ability of edible plants to improve antioxidant capacity and decrease plasma MDA concentration. Accordingly, Micallef et al. [48] demonstrated that ingestion of 400 mL/day of red wine enriched with polyphenols significantly increased plasma thiol and decreased plasma MDA in young and old subjects, suggesting that polyphenols promoted the protection of both lipid and protein oxidation to avoid oxidative damage of the arterial walls and oxidative complication. Other authors have found that polyphenol-rich antioxidant containing pomegranate extract and green tea significantly reduced plasma MDA with increased antioxidant defense, indicating that supplementation of polyphenol-rich antioxidants has important against effects on oxidative stress and lipid peroxidation in type 2 diabetic patients [49]. Similarly, consumption of foods rich in polyphenols with high carbohydrate diet have been reported to decrease lipid and protein oxidation in overweight subjects [44]. Thus, increased antioxidant capacity and decreased lipid peroxidation observed after CTE ingestion may be related to polyphenols containing in CTE which has an in vitro antioxidant activity such as 1,1-diphenyl 2-picrylhydrazyl (DPPH), hydroxyl radical scavenging activity (HRSA), superoxide radical scavenging activity (SRSA), FRAP and TEAC [21]. It is assumed that antioxidant activity of CTE is at least in the part directly responsible action for reduced oxidative imbalance mediated by sucrose.

Interestingly, it has demonstrated that consumption of sucrose and fructose have been linked to increased plasma uric acid [50]. In this pathway, uric acid is produced from fructokinase-mediated metabolism to fructose-1-phosphate [50]. It is well recognized that increased plasma uric acid may result in augmentation of plasma antioxidant potential by increasing FRAP level [17]. In our study, the sucrose load increased in postprandial plasma uric acid and was paralleled by a rise in plasma FRAP levels at 60 min. This is consistent with the previous study of Lotito et al. [51] reporting the rise of FRAP after consumption due to sucrose induced-generation of uric acid in healthy subjects. However, ingestion of CTE and sucrose resulted in higher FRAP level without any change in plasma uric acid. Therefore, the rise of plasma FRAP level after consumption of CTE is not due to the sucrose load and its elevating effect on the plasma level of uric acid. The main limitation of this study is that the impact of CTE was observed in healthy men. These results cannot be generalized to all population, in particular to those with glucose intolerance, type 2 DM or different sex and ages.

## Conclusions

Consumption of CTE beverage increases plasma antioxidant capacities without hypoglycemia in healthy subjects. Furthermore, CTE reduces postprandial plasma glucose and insulin concentration concomitant with improved antioxidant status in the subjects when consumed with sucrose. Future research will concentrate on how CTE may be used to modulate glycemia when co-ingested with complex carbohydrates such as white rice and bread. Any positive results that emerge from such studies will enable us to provide a public health advocacy on how such simple food based interventions may be used in our war against diabetes.

## Abbreviations

ABTS: 2,2′-azinobis(3-ethylbenzothiazoline-6-sulfonate)
ALT: Alanine aminotransferase
AST: Aspartate aminotransferase
BHT: 2,6-di-tert-butyl-4-methylphenol
BMI: Body mass index
BUN: Blood urea nitrogen
CTE: *Clitoria ternatea* flower extract
DPPH: 1,1-diphenyl 2-picrylhydrazyl
FRAP: Ferric-reducing ability of plasma
HRSA: Hydroxyl radical scavenging activity
iAUC: Incremental area under the curve
MDA: Malondialdehyde
NCDs: Non-communicable diseases
ORAC: Oxygen radical absorbance capacity
PBS: Phosphate buffer saline
PUFA: Polyunsaturated fatty acids
ROS: Reactive oxygen species
SRSA: Superoxide radical scavenging activity
TBA: Thiobarbituric acid
TBARS: Thiobarbituric acid reactive substances
TCA: Thichloroacetic acid
TEAC: Trolox equivalent antioxidant capacity
